# Characteristics of the Gut Microbiota in Regard to Atopic Dermatitis and Food Allergies of Children

**DOI:** 10.3390/biomedicines12030553

**Published:** 2024-03-01

**Authors:** Alexandra I. Nekrasova, Irina G. Kalashnikova, Maria M. Bobrova, Anna V. Korobeinikova, Sirozhdin Yu. Bakoev, German A. Ashniev, Ekaterina S. Petryaikina, Alexander S. Nekrasov, Angelica V. Zagainova, Mariya V. Lukashina, Larisa R. Tolkacheva, Anastasia S. Zhdanova, Vladimir E. Mukhin, Vladimir S. Yudin, Anton A. Keskinov, Valentin V. Makarov, Sergey A. Kraevoy, Sergey M. Yudin

**Affiliations:** Federal State Budgetary Institution “Centre for Strategic Planning and Management of Biomedical Health Risks” of the Federal Medical and Biological Agency, Pogodinskaya Str. 10/1, 119121 Moscow, Russiambobrova@cspfmba.ru (M.M.B.); gashniev@cspfmba.ru (G.A.A.); mlukashina@cspmz.ru (M.V.L.);

**Keywords:** gut microbiota, microbiome, microbial diversity, food allergy, atopic dermatitis, atopy, dysbiosis, 16S rRNA

## Abstract

The gut microbiota plays an important role in maintaining human health, as well as in the development of various pathologies, as indicated by a large amount of research. One of the manifestations of an imbalance in the gut microbiome composition is the appearance of various diseases or immune reactions, in particular, atopic dermatitis (AD) and/or food allergies (FA). In this research, using 16S NGS sequencing, it was found that the gut microbiome of children with food allergies and children with atopic dermatitis can be characterized as having higher inflammatory potential. Both groups exhibited an abundance of representatives from the *Pasteurellaceae* and *Erysipelotrichaceae* families, as well as a decrease in the relative number of representatives from the *Barnesiellaceae* family compared to healthy participants. In the group of participants with food allergies, there was a decrease in the relative number of *Desulfovibrionaceae* representatives and *Bifidobacteriaceae* family enrichment in relatively healthy participants. In addition, when comparing this group with patients with atopic dermatitis, it was revealed that a number of representatives of such families as *Erysipelotrichaceae*, *Ruminococcaceae* and *Sutterellaceae* prevailed. This information confirms that AD and FA correlate with changes in the composition of the gut microbiota. Further research is needed to determine the cause–effect connections and the effect of compounds derived from the microbiota on the AD and FA development and progression, as well as to create new probiotic drugs to prevent and modulate immune responses, including at an early age.

## 1. Introduction

In recent decades, the amount of allergic reactions among the child population has increased. The most common manifestations of immune system disorders are food allergies and atopic dermatitis, which often occur together [1]. According to international statistics, the prevalence of these conditions is 10–40% in the general population, and there is a tendency to further increase the number of patients with this immune pathology [2,3]. Appropriate programs are being implemented in the healthcare system of Russia due to the increasing number of cases, which is in line with the global trend [4].

Immunoreactive conditions, such as atopic dermatitis, food allergies, allergic rhinitis, and allergic asthma, are characterized by an increase in the secretion of Th2 cytokines. This leads to a higher production of immunoglobulin E (IgE) and eosinophilic inflammation [5,6,7]. Research has demonstrated that children with food allergies and/or atopic dermatitis are more likely to develop asthma later in life [8,9,10]. This is known as the ‘atopic march’ [11].

Atopy, including atopic dermatitis and food allergies, is characterized by a Th2-type immune response. This is likely due to dysfunction of the intestinal barrier caused by disruption of the early colonization processes of symbiotic intestinal bacteria and/or intestinal dysbiosis [12,13,14,15]. Previous studies have demonstrated that a ‘healthy’ microbiota is crucial for the maturation of immune tissues and modulation of immune responses from birth. This mechanism is also essential for the development of immunological tolerance [16]. Disruption of the early colonization process by symbiotic bacteria can lead to the colonization of the intestine by pathogenic microorganisms, which can result in various immuno-mediated pathologies [7]. Research has indicated that dysbiosis during early development may be linked to the development of atopy later in life [17]. As the development of atopic conditions is a complex process, it is necessary to study various factors that may affect the atopic march. This includes examining the development of the Th2-type immune response and investigating any violations of the mechanisms of immune tolerance [18,19]. In addition, the gut microbiota plays a crucial role in maintaining immune homeostasis throughout life by means of microbial components and metabolites. This highlights the interplay between the gut microbiota and immunity [20]. Several studies have established a strong correlation between gut microbiota imbalance and susceptibility to immune disorders, as well as the onset of atopic diseases like atopic dermatitis, asthma, and food allergies [20]. Intestinal dysbiosis may cause disorders in regard to intestinal barrier function, which can have a systemic effect on immune function and contribute to the development of immune-mediated pathologies [13,21]. Previous studies have shown that patients with atopic dermatitis and food allergies exhibit an imbalance in their intestinal microbiota. This imbalance is characterized by a reduction in bacterial diversity and a decrease in the number of certain beneficial bacterial taxa, such as *Lactobacillus* and *Bifidobacterium*, as well as an increase in potentially pathogenic bacteria, such as *Clostridium difficile* [13,22,23,24]. Our current understanding of the atopic march indicates that preventing atopic dermatitis and/or food allergies in early childhood may serve as a preventive measure against the development of asthma later in life [18,19]. Therefore, it is crucial to expand our knowledge of the influence of intestinal microbiota on the development of atopic conditions, which can lead to new therapeutic approaches [25].

## 2. Materials and Methods

### 2.1. Description of the Studied Groups

This research was conducted in accordance with the guidelines of the Helsinki Declaration and approved by the Committee on Institutional Ethics of the Federal State Budgetary Scientific Institution “Federal Research Centre of Nutrition, Biotechnology and Food Safety”, Moscow, Russia (protocol No. 5, 2 July 2020). All data were obtained from a cohort of patients with atopic dermatitis and food allergies in the autumn–winter period (October–November) living in the Moscow region, for whom the criteria for inclusion in this analysis were met. Patients and legal representatives of juveniles were invited to participate in the study, and all procedures and possible risks were explained. If consenting, legal representatives of juvenile patients signed the appropriate informed consents, as well as consented to personal data processing. Healthy volunteers formed a control group. The ages of the children ranged from 3 to 12 years (Table 1).

Participants meeting the following inclusion criteria were included in the research:

- Children aged 3–12 years with a confirmed diagnosis of atopic dermatitis and food allergies;
- The opportunity to take fecal and blood samples, which does not affect the current state of the patient’s health;
- Absence of genetic, hereditary and hematological disease history;
- Absence of infectious diseases in the acute phase, absence of chronic gastrointestinal diseases, cardiovascular system, renal disorders, respiratory diseases, endocrine disorders, oncological diseases, autoimmune diseases, no drug intake (including probiotics) on a regular basis, absence of taking antibiotics (including other antibacterial drugs) and probiotics in the last two months;
- Availability of the necessary documents signed by the child’s parent or legal representative; informed consent to participate in the research; informed consent to the processing of personal data with full knowledge and understanding; informed consent to medical manipulations with full knowledge and understanding.

All participants underwent a survey, physical examination, and the collection of blood and fecal samples. Blood serum samples were collected once during the initial examination of the participant by venipuncture into vacutainers with K3-EDTA (Becton Dickinson, Franklin Lakes, NJ, USA). Fecal samples were collected during the initial examination of the patient after inclusion in the study in accordance with the inclusion/non-inclusion criteria. All participants did not receive any antibiotics or purgatives a month before the research.

The first group consisted of 150 people. Of these, 50 people with AD, 50 people with FA, and 50 people were included in the control group (control). Fecal samples were donated by patients and healthy volunteers who signed an informed consent. After sampling, the specimens were stored at a temperature of +4–+6 °C for a maximum of 2 h before being transferred to the laboratory for long-term storage at a temperature of −80 °C. During transportation, the samples were kept in a special container with dry ice at a temperature no higher than −70 °C.

### 2.2. Measuring the Level of Total Immunoglobulin IgE

The measurement of the total level of IgE in blood serum was performed by the ELISA method using a kit for the quantitative determination of human IgE (FineTest, Wuhan, China) according to the manufacturer’s instructions. Optical density was measured at a wavelength of 450 nm on a tablet reader Infinite F50 (Tecan, Grödig, Austria). The concentration was measured in ng/mL, and all measurements were carried out in duplicate using standard solutions.

### 2.3. DNA Isolation and Libraries Preparation

QIAamp DNA Stool Mini Kit (Qiagen, Hilden, Germany) was used for bacterial DNA isolation from fecal samples. All procedures were carried out according to the manufacturer’s instructions. Quality and quantity of DNA were estimated by the dsDNA HS Assay Kit on a Qubit^®^ 4.0 fluorimeter (Thermo Fisher Scientific, New York, NY, USA) and by electrophoresis in 1% agarose gel.

Metagenomic analysis of intestinal microbiota samples was performed using high-throughput sequencing (NGS) by targeted sequencing, followed by bioinformatic analysis of the nucleotide sequence of the variable sections of the V3–V4 16S rRNA gene. The procedures were strictly carried out in accordance with the manufacturer’s instructions. The quality of the prepared libraries was analyzed via the Bioanalyzer 2100 device (Agilent Technologies, Santa Clara, CA, USA) using a Highly Sensitive DNA kit (Agilent Technologies, Santa Clara, CA, USA). Libraries were prepared using MiSeq V2 Nano and Nextera XT Index Kit (Illumina, San Diego, CA, USA). The samples were sequenced using the Illumina MiSeq platform (Illumina, San Diego, CA, USA) according to the manufacturer’s protocol, utilizing reagents for two-sided reading and a reading length of at least 250 bp. The amount of PhiX Control v3 was at least 1%.

### 2.4. Processing and Analysis of Metagenomics Data

Quantitative Insights in Microbial Ecology 2 (QIIME2), version 2022, was used to analyze and process the obtained sequencing data [26]. 

Using the cutadapt plug-in implemented in the QIIME2 toolkit, primer residues (flanking areas V3–V4) that could potentially introduce contamination to the Illumina sequencing data were removed [27]. Subsequently, the processed reads underwent trimming, filtering, and merging using DADA2 [28]. This resulted in the generation of a table containing all the detected amplicon sequence variants (ASVs) in each sample, along with their respective absolute and relative abundances. A phylogenetic tree with a midpoint root was constructed using the FastTree plugin (v2023.5.0) [29], employing multiple sequence alignments facilitated by MAFFT (v7.453) [30]. The taxonomic label assignment for each ASVs was performed using the Naive-Bayes classifier from the Python scikit-learn library. The classifier was trained on the 16S rRNA reference databases SILVA (v.138.99) and RDP (v.11.5). The resulting taxonomic tables were then merged with the patient’s meta information and ASV data. A subsequent taxonomic analysis was conducted at the family level.

### 2.5. Statistical Analysis

#### 2.5.1. Program Software

All calculations were performed using the R programming language (version 4.2.1), with average data processing by R-Studio 2022.02.3 (build 492).

#### 2.5.2. Data Transformation

In order to mitigate the impact of sampling bias caused by underrepresented families, a customized normalization procedure was implemented. This procedure aimed at prioritizing families based on a specific criterion. To achieve this, any values in the ASV (amplicon sequence variant) frequency table below 31 were substituted with 0, values between 31 and 99 were replaced with 75, and values ranging from 100 to 149 were replaced with 125. Values outside these predefined ranges were left unaltered. This approach was instrumental in addressing the underrepresentation of certain families in the dataset, thus reducing the impact of sampling bias. Furthermore, the Next Generation Sequencing (NGS) data underwent conversion to the phyloseq format using the phyloseq (v. 1.46.0) package in R, which employs a specialized S4 class system. This conversion process facilitated the organization and analysis of the sequencing data, enabling a more comprehensive and standardized approach to data interpretation and comparison.

In addition to the normalization procedure, sequencing depth control measures were employed to ensure the accuracy and reliability of the data. This involved the evaluation of 16S rRNA coverage in the initial 150 samples dataset. To avoid rarefaction bias, a coverage threshold was specified at the 20k level, resulting in 128 samples that passed the empirical threshold. By implementing this approach, the sequencing depth control effectively minimized the impact of rarefaction bias, ensuring that the dataset was representative and robust for subsequent analysis and interpretation.

#### 2.5.3. Alpha Diversity

In our investigation of sample richness and the assessment of differences between the analyzed groups, a comprehensive approach was undertaken. We calculated six alpha-diversity indices, including Shannon, Pielou, Chao1, Simpson, Faith, and Strong, for samples from each analyzed cohort. These indices provided a multifaceted understanding of the biodiversity within the respective groups. To visually represent the intricate biodiversity patterns observed, scale diagrams were generated for the three distinct groups, offering a comprehensive overview of the sample richness and diversity. A pairwise comparison of samples was conducted for each taxon using the Mann–Whitney criterion, enabling a detailed examination of the differences between the samples. A statistical assessment was performed on the three groups using the Kruskal–Wallis criterion, providing valuable insights into the group disparities. The resulting *p*-values were calculated with adjustment for a False Discovery Rate (FDR) to effectively mitigate the occurrence of false positive outcomes, ensuring the reliability and accuracy of our findings. All calculations were performed using the stats package in R (v.4.2.1).

#### 2.5.4. Beta Diversity

The microbiome data underwent normalization utilizing the log10x formula to ensure a standardized and comparable framework for analysis. Subsequently, a canonical redundancy analysis was conducted by leveraging the distance matrix, facilitated by the vegan package (v.2.6-4). The rda() function within the package was employed for this purpose, enabling a comprehensive assessment of the relationships and patterns within the data. Furthermore, a multivariate analysis of variance was executed using the manova() function from the stats package (v.4.2.1) to ascertain the existence of dissimilarities among the various groups, providing valuable insights into the variations and relationships present within the dataset.

#### 2.5.5. Linear Discriminant Analysis Effect Size (LEfSe)

In order to compute the effect size of linear discriminant analysis (LDA) using phyloseq format data, the LEfSe package (Linear discriminant analysis Effect Size) [https://www.bioconductor.org/packages/release/bioc/html/lefser.html (accessed on 5 September 2023)] was utilized. This tool identifies the features that are most likely to explain the differences between the groups presented by combining standard tests for statistical significance with additional tests encoding biological consistency and the relevance of effects. Studies were conducted with default parameters.

#### 2.5.6. Differential Population Analysis

The objective of this step is to ascertain the specific taxa that exhibit alterations in microbiota composition during the course of the disease. Each bacterial family was evaluated separately in pairs between groups. In order to account for the non-normal distribution of the number of Amplicon Sequence Variants (ASVs), a nonparametric Mann–Whitney test was conducted using the stats package (v.4.2.1). To mitigate the risk of false positives, *p*-values were calculated with a false discovery rate (FDR) adjustment.

#### 2.5.7. Regression Tree

The rpart package (v.4.1.23) was employed to establish a linkage between the dependent and independent variables, enabling the formation of a binary tree. The rpart() function was specifically utilized for this purpose. To optimize the binary tree by removing unnecessary branches, the prun() function was applied. Furthermore, the DMwR package (v.0.4.1) was utilized for visualization purposes, utilizing the prettyTree() function. To enhance the accuracy of the model and minimize the influence of noisy data, predictor variables that contributed to the noise were eliminated. Specifically, collinear variables with a cutoff of 0.4 were excluded from the analysis, as well as variables exhibiting near-zero variance. The best tree was considered to be the tree consisting of such a number of branches t for which the sum (Cross-validated error (CV_er_) + Standard error (SE)) was the minimum.

#### 2.5.8. Binary Classification

Binary classification was used for paired comparison of groups. The preprocessing was carried out similarly to the previous stage, with the data being divided into training and test data in a ratio of 80:20. The model used a training dataset and calculated how to match the input data with class labels. The efficiency of classification models with the pROC package (v.1.18.5) by the roc() function was compared. Evaluation of the results of the ROC analysis gave us reason to believe that Random Forest is the most accurate option for the presented data, and this technique was further used for the intergroup analysis (Random Forest package) [https://CRAN.R-project.org/doc/Rnews/ (accessed on 5 September 2023)].

## 3. Results

After analyzing the quality of the series, 128 samples (49 AD, 47 FA and 32 control) were included in the final metagenomics analysis. A nonparametric statistical Mann–Whitney test was performed for data on alpha-diversity. 

**Figure 1:**
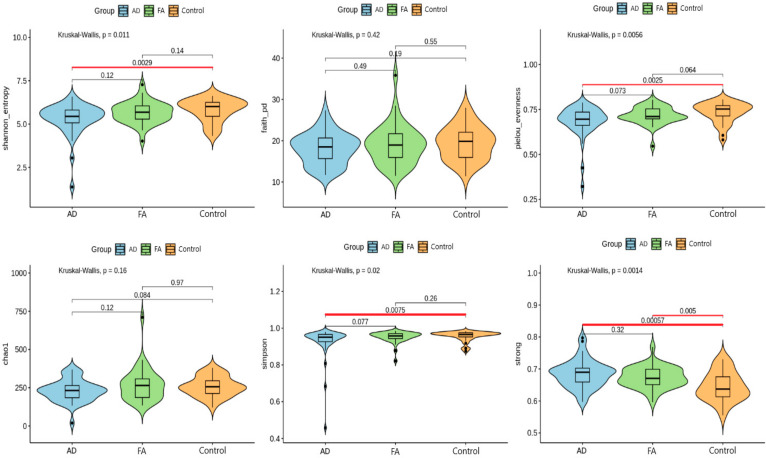
Statistical analysis results for alpha diversity values. The *p*-value values are represented above each line. (AD—atopic dermatitis, FA—food allergy, control—healthy people).

The assessment of the alpha diversity of microbial communities (Figure 1) was carried out using six indices (Shannon, Pielou, Chao1, Simpson, Faith, and Strong) for all groups of samples to assess the representation and uniformity of the composition of the intestinal microbiota. The figure displays statistical analysis results for alpha-diversity values, comparing the distribution of alpha-diversity indices among different cohorts. Grouped violin plots correspond to alpha-diversity indices labeled on the Y-axis. Significant differences between cohorts are highlighted in red, and corresponding *p*-values are shown above each connecting line. Significant differences were observed (*p*-value < 0.05) between the groups with atopic dermatitis and the control group for the Shannon, Pillow, Simpson, and Strong indices. The participants of the group with food allergies had significant differences in the diversity of the microbiota compared to healthy ones according to the Strong index (*p* = 0.005). The groups with atopic dermatitis and food allergies had the same enrichment and uniformity of the intestinal bacterial community (when comparing groups with atopic dermatitis and food allergies, there were no significant differences between them).

**Figure 2:**
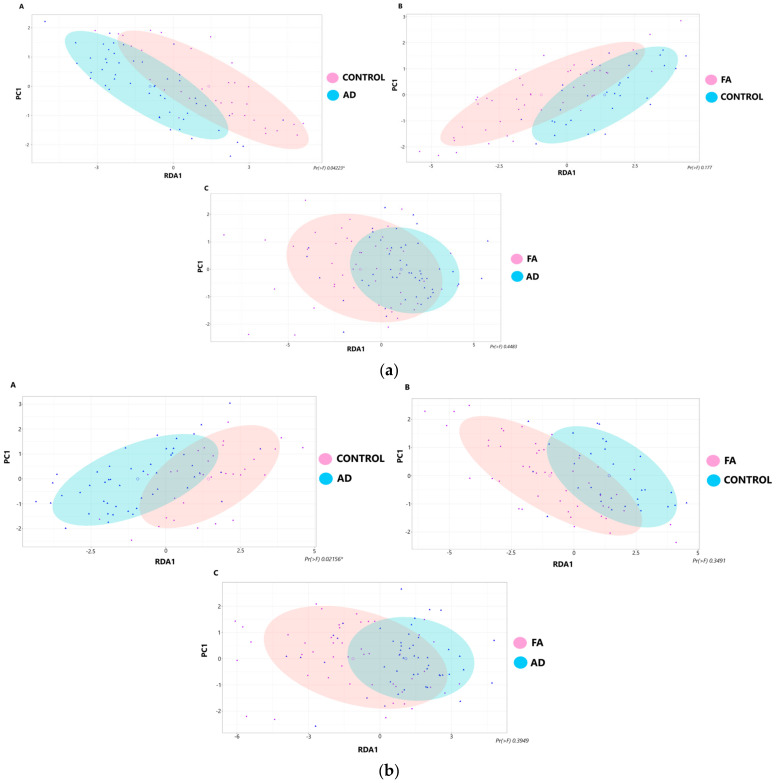
Results of the β-diversity analysis. (**a**) Ordinal analysis of groups for RDP data (**b**) Ordinal analysis of groups for SILVA data (*—significant differences).

The degree of intergroup dissimilarities in beta diversity was assessed using a redundancy analysis (RDA). Figure 2 shows the results of the β-diversity analysis (the ordination diagram in coordinates {RDA1, PC1} is used). The figure presents the results of a β-diversity analysis, specifically focusing on the comparison of groups using RDP and SILVA databases. The analysis was conducted in an ordinal manner, and the figure consists of two panels: (a) ordinal analysis of groups for RDP data, and (b) ordinal analysis of groups for SILVA data. The figure employs a pairwise comparison approach to showcase the results obtained from the RDP and SILVA databases. Significant differences in the results of permutation tests of variance were noted only for the AD group (*p*-value < 0.05) relative to the control group.

**Figure 3:**
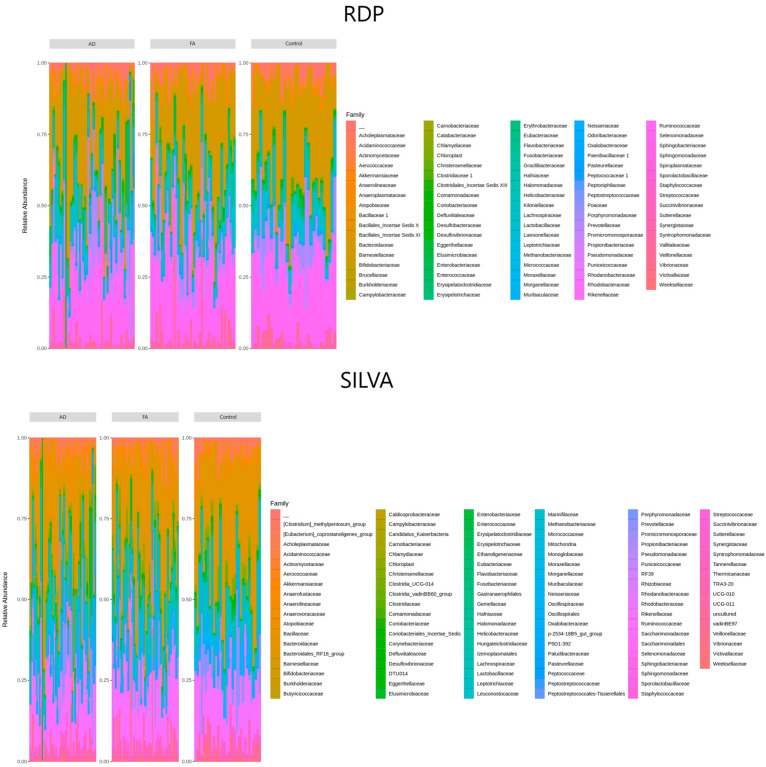
Relative abundance of bacteria.

The composition of microbial communities of the studied groups was then analyzed at the family level in accordance with the protocol given in Section 2.5.2. «Data Transformation». The ASV set consisted of 89 families from the RDP table and 111 SILVA families (Figure 3). We used two curated databases SILVA and RDP in order to cross-validate obtained taxonomic assignments and access sustainable results. The figure presents barplots depicting the relative abundance of bacteria obtained from a 16S rRNA analysis using QIIME2. The barplots provide a visual representation of the taxonomic composition of the gut microbial community in each cohort. Each bar represents the proportion of a specific bacterial taxon within the analyzed samples and provides a snapshot of the microbial composition, helping identify dominant or rare taxa within the community.

**Figure 4:**
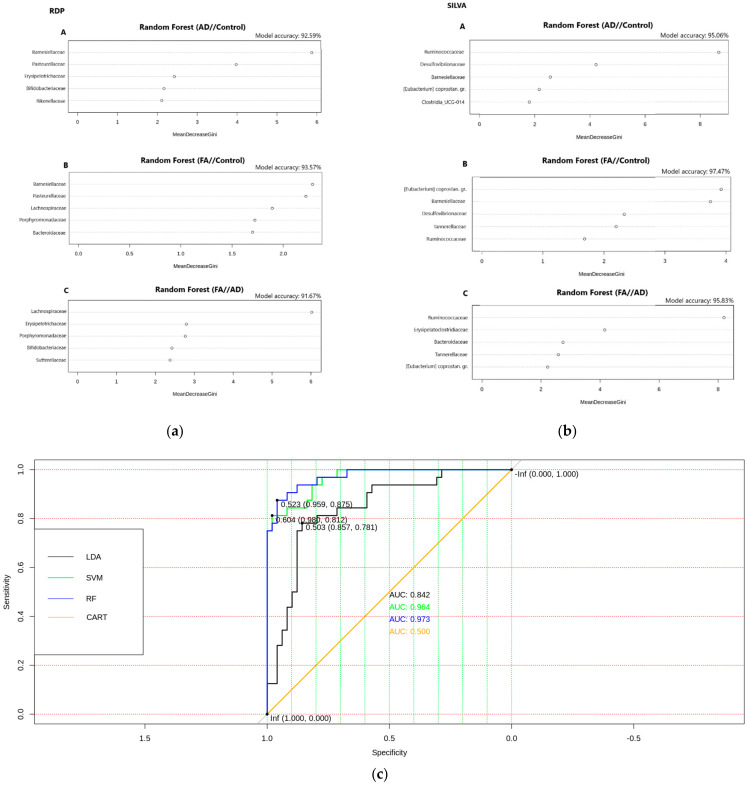
The results of the Random Forest model (**a**) for RDP data, (**b**) SILVA data (**c**), ROC curves for four classification models: LDA, SVM, RF, CART for constructing an optimal binary classification model. The best accuracy estimation result was achieved via the Random Forest model.

The following are the results of the analysis of 16S rRNA sequencing for statistically significant families found in the RDP and SILVA databases based on the results of the LEfSe (LDA effect size) analysis and the Random Forest model (Figure 4). The visual representation of the model performance highlights the superior accuracy achieved by the Random Forest model in this binary classification task for two distinct datasets: related either to RDP (a) or SILVA (b) databases. The figure includes Receiver Operating Characteristic (ROC) curves for four classification models: Linear Discriminant Analysis (LDA), Support Vector Machines (SVM), Random Forest (RF), and Classification and Regression Trees (CART). The ROC curves illustrate the performance of each classification model in terms of sensitivity and specificity across various classification thresholds. These curves provide insights into the trade-off between true positive rates and false positive rates for each model. The Random Forest model achieved the highest accuracy estimation result when compared to the other classification models. This indicates that the Random Forest model outperformed LDA, SVM, and CART in accurately classifying the binary outcomes.

Bacterial families showing significant results were additionally tested by the Mann–Whitney criterion adjusted for FDR; the results are presented in Table 2.

Table 2 demonstrates the comparison of the microbial composition of the intestine of the control group with the group of patients with AD. It is noted that the number of representatives of the *Pasteurellaceae* family increases in the AD group and the number of representatives of the *Barnesiellaceae* family decreases.

In comparing the group with FA against the control group, there was a decrease in the number of representatives of the families *Barnesiellaceae* and *Desulfovibrionaceae*, as well as an increase in the number of representatives of the families *Bifidobacteriaceae*, *Ruminococcaceae*, and *Erysipelotrichaceae* in the group with FA.

In comparing the group with AD against the FA group, there was an increase in the number of representatives of the families *Sutterellaceae*, *Ruminococcaceae*, and *Erysipelotrichaceae* in the group with FA.

**Figure 5:**
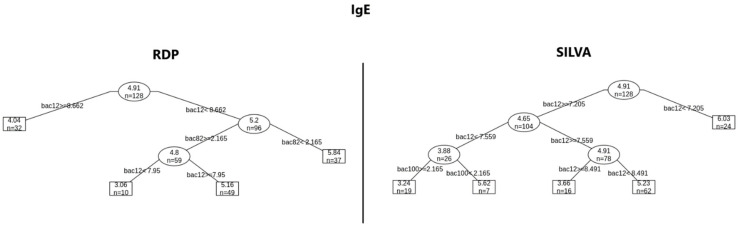
Regression tree for IgE as an independent variable (*Ruminococcaceae* and *Sutterellaceae* (RDP: bac12 and bac82; SILVA: bac12 and bac100, respectively).

IgE levels were measured for samples from all patient groups, as it is an important clinical indicator for the diseases under consideration. Then, the correlation of the IgE level with the representation of families was analyzed (Figure 5). The root node represents the mean IgE value, and the branches connecting the nodes represent the found parameters of the data partitioning. The model starts with the entire dataset and tries all the different values of each input variable to find a predictor and partition value that separates the data into two regions. After finding the best partition, the data is split into the two resulting groups and the partitioning process is repeated for each of the two regions. This process continues until some stopping criterion is reached. Thus, it is easy to understand which variables are important for forecasting. Based on our graph, in the first step, when analyzing the whole sample, the best predictor for partitioning was *Ruminococcaceae* (bac12 RDP, bac12 SILVA). In subsequent steps, the family *Sutterellaceae* (bac82 RDP, bac100 SILVA) proved to be the best variable for partitioning. Since the bac12 data show opposite types of correlation with IgE levels at partitioning and further reduction of groups, we can assume that the mean bacterial level in our data (7.95 < x < 8.662) is related to the mean IgE (4.91–5.16), as outliers cause fluctuations in bacterial levels. The family *Sutterellaceae* showed a more unambiguous result: the IgE level was higher when this family was reduced. Thus, it was shown that the representation of the *Ruminococcaceae* and *Sutterellaceae* families in the studied samples was associated with the IgE level.

## 4. Discussion

Changes in the biodiversity of the host-gut microbiome and its metabolic activity have been associated with greater susceptibility to immune-mediated disorders, including allergic diseases [13,21]. The development and balance of the immune system is influenced by interactions between commensal bacteria, intestinal epithelial cells of the host, and immune cells [12,13,20]. Several studies have shown a close relationship between changes in the diversity and composition of the intestinal microbiota [20] and the development of allergic diseases, such as asthma, food allergies, and atopic dermatitis. For instance, research has shown that patients with atopic dermatitis have a higher proportion of *Clostridium difficile*, *Escherichia coli*, and *Staphylococcus aureus* in their intestinal microbiome compared to healthy individuals. Additionally, the proportion of *Bifidobacteria*, *Bacteroidetes*, and *Bacteroides* is relatively lower in these patients [31]. These findings are in line with research on food allergies, where some authors have observed that certain Clostridia species in the gut microbiota can stimulate the production of Tregs, which reduces the body’s ability to suppress allergic inflammation and oral tolerance [22]. Additionally, Watanabe et al. found that patients with atopic dermatitis had a lower number of *Bifidobacterium* representatives in their gut microbiota compared to healthy children. Furthermore, the quantity and proportion of bifidobacteria varied depending on the disease condition, with lower levels being observed in patients with severe atopic dermatitis [32]. Research has demonstrated that children with IgE-mediated food allergies exhibit a reduction in microbial diversity in relatively healthy groups, with increased levels of representatives from the genera *Clostridium* and *Anaerobacter* and relatively decreased levels of representatives from the genus *Bacteroides* [15,33]. A recent large prospective study [34] found that children who developed blood pressure at school age had lower representations of the genera *Lachnobacterium* and *Faecalibacterium* compared to healthy children in the control group. Studies of fecal samples from children in Nordic countries have shown that those with a high number of *Clostridium* and *Staphylococcus aureus* representatives are more prone to allergic reactions, while healthy children have a predominance of *Lactobacillus* representatives [35,36]. In one of the largest prospective cohort studies of KOALA [37], it was observed that a high relative number of *Escherichia coli* and *Clostridium difficile* representatives is associated with an increased risk of developing atopic dermatitis. Additionally, researchers have noted a correlation between low diversity of the intestinal microbiota, a decrease in short-chain fatty acids, and the development of atopic conditions in children [38]. This suggests that the abundance, diversity, and metabolic activity of the intestinal microbiota are crucial to maintaining homeostasis and good health.

The abundance, diversity and metabolic activity of the intestinal microbiota play an important role in maintaining homeostasis in a healthy organism. Microbial intestinal disorders affect the pathogenesis of allergic diseases, including asthma, FA, and AD, through an aberrant immune response [39].

The investigation of the microbiota composition in three distinct participant groups, employing multiple statistical analysis methods, revealed significant dissimilarities in the taxonomic composition of the intestinal microbiota among all groups when compared to one another. It was found that the gut microbiome of children with FA and children with AD is characterized by a large number of bacterial families associated with inflammatory potential and a generally low diversity of intestinal microbiota. This is indicated by the abundance of representatives of the family *Pasteurellaceae* and a decrease in the relative number of representatives of the family *Barnesiellaceae* in the AD and FA groups relative to the control group. At the same time, there is an increase in the representation of the family *Erysipelotrichaceae* when comparing the FA group with both the AD group and the control group. In the FA group, there was a decrease in the relative number of representatives of *Desulfovibrionaceae* and enrichment of the families *Ruminococcaceae* and *Bifidobacteriaceae* relative to the control group. In addition, when comparing FA with AD groups, it was revealed that the number of representatives of such families as *Erysipelotrichaceae*, *Ruminococcaceae* and *Sutterellaceae* prevailed in the group of children with food allergies. We also noted that the *Ruminococcaceae* and *Sutterellaceae* families were the most dependent on serum IgE levels.

The predominance of the *Erysipelotrichaceae* and *Pasteurellaceae* communities is consistent with an early study of food allergies [40] in children, as these groups were enriched in children with food allergies when compared with their non-allergic siblings. Chen C.C. et al. observed an increase in the relative number of representatives of *Pasteurellaceae* in children with hypersensitivity to egg whites [41]. Representatives of the family *Pasteurellaceae* have an outer membrane consisting mainly of lipopolysaccharides, which are capable of inducing the attraction and activation of inflammatory cells and the production of pro-inflammatory cytokines [42], which may cause their pro-inflammatory potential. There is evidence that representatives of the family *Erysipelotrichaceae* were previously associated with immune-mediated inflammatory diseases. Patients with multiple sclerosis had an increased number of representatives of this family in the metagenome [17]. In research on mice in the IBD model, representatives of the *Erysipelotrichaceae* were classified as colitogenic representatives of the intestinal microbiota. These representatives were also enriched with IgA, which, according to the authors of the work, contributes to the development of inflammation [43], as well as to an increase in the permeability of the intestinal epithelial barrier. Also, according to the literature data, an increase in the number of representatives of the family *Erysipelotrichaceae* is associated with a high level of trimethylamine N-oxide, the proinflammatory and proatherogenic activity of which has been shown in a number of analyses, as well as the association with metabolic disorders [44,45,46].

The number of representatives of the *Barnesiellaceae* family was reduced in comparison with healthy people, which has been noted in several research studies. For example, in [47], the content of *Barnesiellaceae* was lower in patients with cardiovascular diseases. In earlier studies, it was noted that a decrease in the relative number of representatives of this family was associated with autoimmune processes in the body, such as Behcet’s disease [48]. In [49], a positive correlation was observed between the relative number of representatives of the *Barnesiellaceae* family and TNF-α levels in HIV patients. A study on mice [50] found an association between a decrease in the number of *Barnesiellaceae* and the development of colitis; this may indicate that a decrease in the number of representatives of this family can potentially affect the development of inflammation in atopic dermatitis and food allergies.

In the group of children with food allergies, the levels of proteobacteria *Sutterellaceae* were increased. This family has previously been associated with other inflammatory diseases, such as Crohn’s disease and ulcerative colitis, and it has also been found in healthy adults [51,52]. In addition, associations in this family with atopic dermatitis in children were identified in Chinese research [53]. However, it is still not entirely clear whether members of the family *Sutterellaceae* are involved in inflammation or are part of the normal human gut microbiome.

A small amount of bacteria from the family *Desulfovibrionaceae* is part of the normal intestinal microbiota and belongs to sulfate-reducing bacteria of the *Proteobacterium* variety [54]. In our study, there was a decrease in the number of representatives of this group of bacteria in patients with food allergies, which could potentially contribute to the development of food allergies. It was previously noted that the number of representatives of the family *Desulfovibrionaceae* in the microbiome correlates with the fat content in the food consumed [55], which could have affected the results of our research. There is also evidence that representatives of this family are associated with metabolic diseases and inflammation in mouse models [56]. Some research has shown that the abundance of representatives of *Desulfovibrionaceae* is associated with various inflammatory diseases [38], as well as the production of endotoxins, so the results of our research contradict the data presented in world publications. The association of *Desulfovibrionaceae* reduction and food allergy requires further research. The abundance of representatives of the families *Bifidobacteriaceae* and *Ruminococcaceae* in the group with food allergies is also consistent with early research on food allergies in children [33,57]. In addition to allergies to cow’s milk protein, representatives of the family *Ruminococcaceae* were also previously associated with food allergies regarding eggs [58]. Supposedly, the high relative abundance of *Ruminococcaceae* due to metabolic activity disrupts the regulation of effector mast cells of the intestine, thereby increasing its permeability [44]. However, there are studies in which food allergies are characterized by a decrease in the number of representatives of *Bifidobacteriaceae* and *Ruminococcaceae* [59]; additionally, some studies have shown that both food allergies and/or atopic dermatitis are characterized by a decrease in the number of representatives of *Bifidobacteriaceae* and *Ruminococcaceae* [60,61], which is associated with increased TLR2-induced reactions of IL-6 and TNF-α [61].

Atopic dermatitis and IgE-mediated food allergies are characterized by an increase in the total serum IgE level, which was confirmed in our research. We noticed that some bacterial taxons of the gut microbiota, namely *Ruminococcaceae* and *Sutterellaceae*, are associated with an increase in the concentration of this class of immunoglobulins in the blood serum, which may indicate that the families of *Ruminococcaceae* and *Sutterellaceae* are potential biomarkers of IgE-mediated food allergies. Other IgE-associated microbial patterns were also identified, so, in [33], children with IgE-mediated food allergies had a positive correlation between representatives of the family *Clostridiaceae* and serum-specific IgE. Therefore, this phenomenon requires additional investigation, taking into account modern studies of the immune status of atopic patients [62,63].

Despite the probably similar etiology of these diseases (AD and FA) [1,13], we observed differences in the composition of the intestinal microbiota of the participants, which could probably be caused by a change in diet in patients with food allergies due to a refusal to consume certain components of the diet. Nevertheless, there were common patterns in the composition (abundance of representatives of the families *Pasteurellaceae* and *Erysipelotrichaceae* and a decrease in the level of *Barnesiellaceae*, as well as a decrease in diversity), which we believe reflects the relationship between immune changes in atopy and the gut microbiota.

## 5. Conclusions

In the course of this investigation, distinct patterns of variation in the intestinal microbiota were observed among patients with atopic dermatitis (AD), food allergies (FA), and the control group consisting of healthy children. Utilizing 16S rRNA sequencing and employing various statistical methods, it was determined that the AD and FA groups exhibited an augmented abundance of representatives from the *Pasteurellaceae* family, while showing a reduced relative abundance of representatives from the *Barnesiellaceae* family in comparison to the control group. Furthermore, an elevated representation of the *Erysipelotrichaceae* family was observed in the food allergy group in relation to both the atopic dermatitis and control groups. In the food allergy group, a decrease in the relative abundance of representatives from the *Desulfovibrionaceae* family was observed, along with an enrichment of the *Ruminococcaceae* and *Bifidobacteriaceae* families, relative to the control group.

Comparison between the AD and FA groups revealed that the number of representatives from families such as *Erysipelotrichaceae*, *Ruminococcaceae*, and *Sutterellaceae* was predominant in the group of children with food allergies. Additionally, it is noteworthy that both the AD and FA groups exhibited lower diversity in the intestinal microbiota, as indicated by the results of the alpha-diversity assessments, in comparison to the control group. This research also demonstrated an association between the *Ruminococcaceae* and *Sutterellaceae* families, as well as IgE levels changes in the serum of children.

In summary, it can be concluded that both atopic dermatitis and food allergies are characterized by dysbiosis of the gut microbiota, with distinctive bacterial signatures proving specific to each condition. However, further analyses involving larger cohorts of participants is necessary to validate these findings and identify specific bacterial species consistently associated with the pathogenesis of atopic dermatitis and food allergies. Given the intricate nature of the structure, functions, and variability of the intestinal microbiome, it is evident that the interaction between the microbiota and human health is dynamic, thus warranting further research in the field of personalized medicine.

## Figures and Tables

**Table 1:** Characteristics of participants.

	AD n = 49	FA n = 46	Control n = 32
Age (Me [25; 75])	7 [6; 10]	6 [4; 9]	8 [5; 11]
Sex%	43% M, 57% F	48% M, 52% F	44% M, 56% F
IgE level (Me [25; 75])	186.36 [94.93; 534.12]	123 [27.8; 579.33]	241.8 [31.73; 444.31]

**Table 2:** Comparison of detected significant bacteria for the studied groups. ↓ decrease in the number of representatives of bacterial families; ↑ increase in the number of representatives of bacterial families.

Family	Relative Abundance	RDP (Adj *p*-Value, α ≤ 0.05)	SILVA (Adj *p*-Value, α ≤ 0.05)
1. Comparison of AD and control groups			
*Barnesiellaceae*	↓ AD	0.03	0.03
*Pasteurellaceae*	↑ AD	0.01	0.01
2. Comparison of FA and control groups			
*Bifidobacteriaceae*	↑ FA	0.001	0.001
*Barnesiellaceae*	↓ FA	-	0.04
*Desulfovibrionaceae*	↓ FA	0.035	0.024
*Ruminococcaceae*	↑ FA	-	0.002
*Erysipelotrichaceae*	↑ FA	0.007	0.008
3. Comparison of FA with AD groups			
*Erysipelotrichaceae*	↑ FA	0.007	0.0002
*Ruminococcaceae*	↑ FA	-	0.012
*Sutterellaceae*	↑ FA	0.028	-

## Data Availability

The datasets used and analyzed in the present study are available from the corresponding author on reasonable request.
